# The effects of chemoradiotherapy on recurrence and survival in locally advanced rectal cancers with curative total mesorectal excision: a prospective, nonrandomized study

**DOI:** 10.1186/s12957-017-1275-4

**Published:** 2017-11-22

**Authors:** Erhan Akgun, Serdar Ozkok, Mevlut Tekin, Tayfun Yoldas, Cemil Caliskan, Timur Kose, Bulent Karabulut, Murat Sezak, Nevra Elmas, Omer Ozutemiz

**Affiliations:** 1 Department of General Surgery, Ege University School of Medicine, Izmir, Turkey; 2 Department of Radiation Oncology, Ege University School of Medicine, Izmir, Turkey; 3 Department of Biostatistics, Ege University School of Medicine, Izmir, Turkey; 4 Department of Medical Oncology, Ege University School of Medicine, Izmir, Turkey; 5 Department of Pathology, Ege University School of Medicine, Izmir, Turkey; 6 Department of Radiology, Ege University School of Medicine, Izmir, Turkey; 7 Department of Gastroenterology, Ege University School of Medicine, Izmir, Turkey

**Keywords:** Rectal cancer, Chemoradiotherapy, Preoperative, Postoperative, Recurrence, Survival

## Abstract

**Background:**

There are only two prospective, randomized studies comparing preoperative long-term chemoradiotherapy and postoperative chemoradiotherapy in locally advanced rectal cancer (LARC); however, conflicting results in terms of locoregional recurrence (LR) and survival rates have been reported. This prospective study aims to compare the effects of preoperative versus postoperative chemoradiotherapy on recurrence and survival rates in LARC patients.

**Methods:**

From January 2003 to January 2016, a total of 336 eligible patients who were clinically diagnosed with LARC (T_3_–T_4_ tm or node-positive disease) were prospectively assigned into preoperative chemoradiotherapy (*n* = 177) and postoperative chemoradiotherapy (*n* = 159) groups. The preoperative treatment consisted of 50.4 Gy total dose of radiotherapy (delivered in fractions of 1.8 Gy) and concomitant two cycles chemotherapy of 5-fluorouracil and leucovorin. The patients in the preoperative group underwent curative total mesorectal excision (TME) following long-term chemoradiotherapy. Surgery was performed 8 (range 4–12) median weeks after the completion of the chemoradiotherapy. Similar protocol was administered to the postoperative group 4 weeks after the operation. Four cycles of adjuvant chemotherapy were added to the groups. The primary end points were locoregional recurrences and 5-year cancer-specific, overall, and disease-free survivals.

**Results:**

The mean follow-up period was 60.4 (range 12 to 168) months. Five-year cumulative incidence of locoregional recurrence (LR) was 7.4% in the preoperative group and 13.4% in the postoperative group (*p* = 0.021). Five-year cancer-specific survival (CSS) was 87.5% in the preoperative group and 80% in the postoperative group (*p* = 0.022). Overall survival (OS) was 79.8 versus 74.7% (*p* = 0.064), disease-free survival (DFS) was 75.2 versus 64.8% (*p* = 0.062), and severe late toxicity was 7.4 versus 13.2% (*p* = 0.002), respectively. The rate of patient compliance was higher in the preoperative group (*p* < 0.001).

**Conclusions:**

Preoperative chemoradiotherapy, as compared with postoperative chemoradiotherapy, significantly improved local control, patient compliance, CSS, and late toxicity and suggested a trend toward improved overall and disease-free survival.

## Background

Today, preoperative (long-term) chemoradiotherapy (CRT) and adjuvant chemotherapy (CT) are standard treatment methods for locally advanced rectal cancer (LARC). This treatment protocol has particularly become popular following the results of the CAO/ARO-94 trial [1]. In this prospective, randomized study comparing preoperative long-term CRT and postoperative CRT in Stage II–III rectal cancers with total mesorectal excision (TME), 5-year cumulative locoregional recurrence (LR) was significantly lower in the preoperative group; however, there was no difference in the overall survival (OS) between the groups. Similar findings have been also reported by the same group in the study which provides results of a 134-month follow-up [2].

The NSABP R-03 trial [3] is another study using a similar protocol. In this study, there was no difference in the LR between the groups; however, 5-year disease-free survival (DFS) was significantly higher in the preoperative CRT group. The results of these two prospective, randomized studies are inconsistent in certain aspects.

In this prospective study, we aimed to compare the outcomes of the preoperative long-term CRT versus postoperative CRT on recurrence and survival rates (OS, DFS, CSS). In addition, we aimed to identify prognostic factors affecting these parameters in patients who underwent curative TME for LARC. The secondary aim was to compare the patient compliance, postoperative mortality and morbidity, and CRT-dependent toxic effects between the patient groups.

## Methods

The study population consisted of a total of 336 patients with LARC who underwent curative TME by a single colorectal surgeon (E.A.) at Colorectal Surgery Unit, Ege University, Faculty of Medicine, Izmir, Turkey, between January 2003 and January 2016. The patients who were eligible for the study were prospectively divided into two groups: preoperative CRT (Group 1, *n* = 177) and postoperative CRT (Group 2, *n* = 159). The study was completed in January 2017.

An approval of the local Ethics Committee (Ege University ethical committee approval number 16-11.1/48) was obtained for this study. A written informed consent was obtained from each patient. The study was conducted in accordance with the principles of the Declaration of Helsinki.

### Patient selection and study design

Locally advanced rectal cancer was defined as T_3_–T_4_ tumor and/or N (+) disease (Stage II–III). The patients who were diagnosed of clinical LARC with a multidisciplinary approach (colonoscopy and rigid rectoscopy, computed tomography, pelvic magnetic resonance imaging, and/or endorectal ultrasound) were evaluated in terms of the eligibility criteria for the study. The eligibility criteria were elaborately presented in Table 1. Briefly; the patients with primary LARC who were electively operated with a lower limit located within the first 15 cm from the anal verge and histopathologically diagnosed with an adenocarcinoma and were susceptible to resect curatively were included in the study.

**Table 1:** Inclusion and exclusion criteria

Inclusion criteria 1) Clinic stage II–III cancer (T3–T4 tm and/or N(+) disease) 2) Patients with histologically confirmed adenocarcinoma of the rectum 3) Tumor distal border located within 15 cm from anal verge (as measured by rigid rectoscopy) 4) Standardized TME surgery 5) Tumor must be clinically resectable with curative intent (R_0_ resection must be most likely) 6) Elective operation 7) The patient must consent to be in the study and the informed consent must be signed
Exclusion criteria 1) Clinic stage I and IV cancer disease 2) Patients with malignant disease of the rectum other than adenocarcinoma 3) Palliative resection (R_2_) 4) Recurrent rectal cancer 5) Emergency cases (MBO, perforation) 6) ASA > 3 patients 7) Other previous or concurrent malignancies 8) Any contraindication for radiochemotherapy 9) Previous chemotherapy or radiotherapy to the pelvis 10) Tumor has arisen from chronic inflammatory bowel disease or hereditary polyposis disease

Before inclusion, the necessity of performing CRT due to LARC, the fact that CRT can be performed preoperatively or postoperatively, and the advantages of the methods and literature data were extensively explained to the eligible patients for the study by the surgeon and radiation oncologist, and voluntary participation to the study was offered which were planned in a randomized manner. However, most of the patients were willing to play an active role in the selection of the treatment group, rather than randomly enter a group. Therefore, the surgeon, radiation oncologist, and patient decided together to the treatment group, and the groups were unable to be randomized.

Chemoradiotherapy protocol was defined as follows:

Computed tomography-based-three-dimensional conformal radiotherapy (RT) was used. In the preoperative setting, the gross tumor volume (GTV) was defined as the gross extend of the primary tumor as assessed by physical examination and imaging studies, including all visible perirectal and affected iliac nodes. The clinical target volume (CTV) covered the entire mesorectum and right and left internal iliac lymph nodes for T3 tumors and the right and left external iliac lymph nodes for T4 tumors with an anterior organ involvement. A 1–2-cm margin in the adjacent organs with a gross tumor invasion was added for T4 lesions. Planning target volume (PTV) was defined by adding a 1.5-cm margin to CTV. The total dose was 50.4 Gy with 1.8 Gy/fractions to GTV and 45 Gy to pelvic lymph nodes. In the postoperative setting, the stapler line or perineal scar was included in the high-risk treatment volume. The total dose to lymphatic areas was 1.8 Gy/fractions to 45 Gy and to postoperative bed was 50.4 Gy. If there were any positive or close margins reported, the doses were uptitrated to 54–59.4 Gy.

The 5-fluorouracil 380 mg/m^2^ (IV bolus) and leucovorin 20 mg/m^2^ (IV bolus) were used as concomitant chemotherapeutic agents. They were applied every 28 days (Days 1–4) for two cycles. After the concomitant applications, 5-fluorouracil 425 mg/m^2^ and leucovorin 20 mg/m^2^ every 28 days (Days 1–5) for four cycles were used as adjuvant to CRT. The patients in the preoperative group received adjuvant CT 4 weeks after the operation, while the patients in the postoperative group received adjuvant CT 4 weeks after CRT completion.

### Surgical technique

All patients were operated by a single, experienced colorectal surgeon (E.A.) using standardized open TME technique. Surgery was performed 8 (4–12) median weeks after the completion of the preoperative CRT. En bloc resection (multi-visceral resection [MVR]) was performed without separating adherences in the patients with an adjacent organ invasion or suspected adjacent organ invasion. The patients with gross residual tumors (R_2_ resection) following the operation were categorized as palliative resection and excluded from the study. Double-staple technique was used for all anastomoses, except those with very low localization. Anastomosis level and integrity were monitored using preoperative rectoscopy. Routine protective stoma was used for patients with an anastomosis level of first 5 cm from the anal verge, whereas stoma was not used for patients with an anastomosis > 7 cm.

### Follow-up

The patients were followed up by general surgery (E.A.), medical oncology (B.K.), or radiation oncology (S.O.) clinics every 3 months during the first 2 years following the treatment, once every 6 months between 2 and 5 years, and once every 12 months after 5 years.

The CRT-dependent acute and late toxic effects were graded according to the toxicity criteria of the Radiation Therapy Oncology Group (RTOG) and the European Organization for Research and Treatment of Cancer (EORTC) [4]. The diagnosis of recurrence was made on the basis of imaging studies and cytological analysis or biopsy, where applicable.

*Isolated local (pelvic) recurrence* refers to recurrent tumor developing only in the pelvis (anastomosis, anterior-posterior-lateral pelvic wall or perineal scar).

*Locoregional recurrence (LR)* refers to both pelvic recurrence and distant metastasis (occurring simultaneously or in various periods). In this study, LR also included isolated local recurrences.

### Pathological examination

The resection specimens were macroscopically examined and reported according to the College of American Pathologists (CAP) protocols [5]. The histological typing and differentiation grading were done according to the World Health Organization (WHO) 2010 criteria. The staging was done according to the TNM staging systems, which classifies the depth of tumor invasion (T), presence of regional lymph metastasis (N), and presence of distant metastasis (M) [6]. Only residually viable tumor areas were evaluated for staging purposes, while lakes of acellular mucin, necrotic tumor cells, or acellular fibrosis replacing the lymph nodes were excluded. The resection was defined as R_1_, if the circumferential resection margin (CRM) was ≤ 1 mm or distal resection margin was ≤ 1 cm distance from the tumor.

### Statistical analysis

In order to detect a reduction in the rate of locoregional recurrence from 12% (postoperative group) to 5% (preoperative group) with 80% probability and a 5% significance level, we calculated that a total of 530 patients had to be recruited.

All eligible and volunteer patients were included in the survival analysis with the cumulative incidence rates of local and distant recurrences per intention-to-treat protocol. Overall survival was defined as the time from the start of the therapy to all-cause mortality or the day of last follow-up. Cancer-specific survival was defined as the time from the start of the therapy to cancer-related mortality. Disease-free survival was defined as the time from the start of the therapy to the first recurrence (LR or distant metastasis) or second primary malignancy.

Statistical analysis was performed using the STATA/MP version 11.1 software (Stata Corp LP, College Station, TX, USA). Comparisons among the groups were performed using chi-square tests (nominal variables), *t* tests (numeric variables), or Mann-Whitney tests (ordinal variables). Survival curves were obtained using the Kaplan-Meier method. The effects of categoric covariates on the survival rates were analyzed using the log-rank test, whereas the effects of numeric covariates were done using the Cox regression analysis in the univariate analysis. Hazard ratios were calculated using the Cox regression analysis. The sub-hazard ratio values were determined using the competing-risk regression analysis for some variables (LR or distant metastasis). Forward likelihood ratio method was applied on variables which were significant in the univariate analysis for multiple Cox regression analysis. A *p* value of 0.05 was considered statistically significant with 95% confidence interval.

## Results

### Patients and surgical procedures

**Fig. 1:**
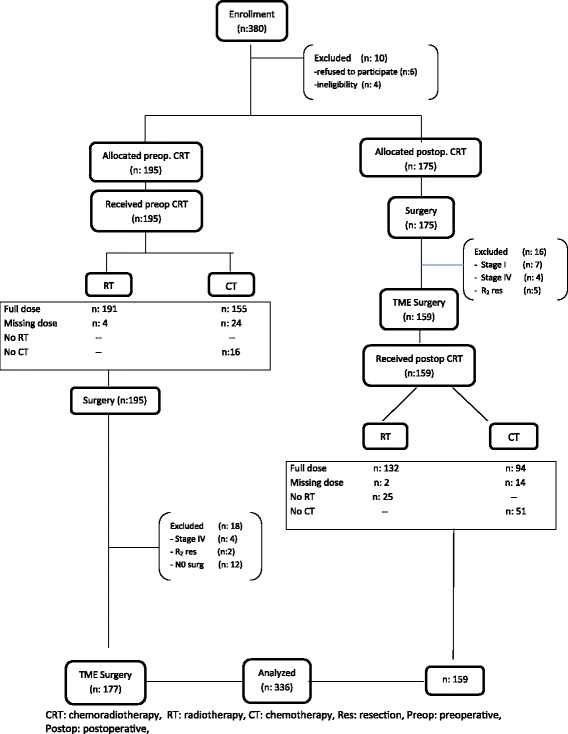
Trial profile

Forty-four of 380 patients were excluded from the study due to various reasons (Fig. 1). A total of 336 patients were evaluated. The baseline characteristics of the patients were similar between the groups (Table 2). Tumor localization was the only significantly different parameter between the groups. The number of lower third rectum tumors was higher in Group 1, whereas the number of upper third rectum tumors was higher in Group 2 (*p* < 0.001). The pre-treatment clinical stages of the groups are shown in Table 2 (baseline characteristics and type of surgery of 336 eligible patients). There was no difference between the groups in terms of clinical stage before treatment (*p*=0.82).

**Table 2:** Baseline characteristics and type of surgery of 336 eligible patients

	Preop. CRT (*n* = 177)	Postop. CRT (*n* = 159)	*p* value
Age (median–range)	61 (22–86)	61 (24–89)	0.88
Sex—no. (%)			0.082
Male	80 (45)	57 (36)	
Female	97 (55)	102 (64)	
Clinical tm stage—no. (%)			0.82
Grade 2	82 (46)	72 (45)	
Grade 3	95 (54)	87 (55)	
Tm localization—no. (%)			*p* < 0.001
Lower third (0 < 5 cm)	97 (55)	52 (33)	
Middle third (5 < 10 cm)	67 (38)	52 (33)	
Upper third (10 < 15 cm)	13 (7)	55 (34)	
Type of resection—no. (%)			*p* < 0.001
AR	10 (5.6)	42 (26.4)	
LAR, intersphincteric	91 (51.4)	67 (42.1)	
APR	72 (40.7)	39 (24.5)	
Total proctocolectomy	4 (2.3)	11 (6.9)	
*MVR resection—*n*			0.11
Total	14	2	
True invasion	8	2	

*MVR multivisceral resection, *APR* Abdominoperineal resection

### Patient compliance

In the preoperative CRT group, 164 patients (92.6%) received a conventional dose of RT and 12 patients (6.8%) received total dose RT at intervals. One patient received a partial dose RT. All patients received RT. In the preoperative group, 142 patients received a conventional dose of CT (80.2%) and 19 patients received partial dose of CT (10.7%), while 16 patients (9.1%) did not receive CT.

In the postoperative group, 116 patients received conventional dose RT (72.9%), 16 patients received total dose RT at intervals (10%), and two patients received partial dose RT. However, a total of 25 patients (15.7%) did not receive RT. Among these patients, 18 patients refused RT and seven patients did not receive RT due to complications. In this group, 94 patients received conventional dose CT (63%) and 14 patients received partial dose CT (9%), while 51 patients did not receive CT (31%) (Fig. 1).

There was a significant difference in the patient compliance between the groups, indicating significantly higher compliance in the CRT group (*p* < 0.001).

### Histopathological results

As shown in Table 3, a significant decrease was seen in the tumor (T), lymph node (N), and stage in the preoperative CRT group.

**Table 3:** Histopathological results of 336 eligible patients

	Preop. CRT (*n* = 177)	Postop. CRT (*n* = 159)	*p* value
R classification—*n* (%)			0.172
R0	163 (92.1)	139 (87.4)	
*R1	14 (7.9)	20 (12.6)	
pT category—*n* (%)			*p* < 0.001
T0	24 (13.5)	–	
T1	10 (5.5)	2 (1.3)	
T2	36 (20.0)	6 (3.8)	
T3	92 (52.0)	133 (83.6)	
T4a	8 (4.5)	16 (9.6)	
T4b	8 (4.5)	2 (1.3)	
pN category—*n* (%)			*p* < 0.001
N0	120 (67.8)	80 (50.3)	
N1a	25 (14.1)	25 (15.7)	
N1b	15 (8.5)	26 (16.4)	
N1c	3 (1.7)	2 (1.3)	
N2a	11 (6.2)	10 (6.3)	
N2b	3 (1.7)	16 (10.1)	
Stage—*n* (%)			*p* < 0.001
**Grade 0/PCR	23 (13.0)	–	
Grade 1	36 (20.0)	–	
Grade 2a	50 (28.2)	74 (46.5)	
2b	7 (4.1)	4 (2.5)	
2c	4 (2.3)	2 (1.3)	
Grade 3a	11 (6.3)	8 (5.0)	
3b	39 (22.0)	53 (33.3)	
3c	7 (4.1)	18 (11.3)	
Differentiation—*n* (%)			0.091
Well differentiation	15 (8.4)	16 (10.1)	
Moderate differentiation	146 (82.5)	114 (71.7)	
Poor differentiation—signed ring cell	7 (4.0)	14 (8.8)	
Mucineous	9 (5.1)	15 (9.4)	
+ PNI—*n* (%)			0.008
Present	10 (5.5)	23 (14.5)	*p* < 0.001
++ LVI—*n* (%)			
Present	3 (1.7)	20 (12.6)	

*+ PNI* perineural invasion, *++ LVI* lenfovascular invasion

*Circumferential resection margin ≤ 1 mm or distal resection margin ≤ 1 cm distance from tumor defined as R_1_ resection

**Pathologic complete response

In terms of the tumor (T), total T_0,1,2_ tumor rate was 39% in Group 1, while it was 5.1% in the postoperative group (*p* < 0.001). The absence of lymph node metastasis (N_0_) was significantly higher in the preoperative group (67.8 versus 50.3%, *p* = 0.002). The rate of Stage III tumor was 32.4% in the preoperative group and 49.6% in the postoperative group (*p* < 0.001).

The rate of R0 resection was higher in the preoperative group (92.1 versus 87.4%), although the difference was not statistically significant. The rate of perineural invasion (PNI) and lymphovascular invasion (LVI) were significantly higher in the postoperative group.

### Postoperative mortality and morbidity

Postoperative mortality (within the first 30 days) was seen in three patients (0.9%). Two patients died due to myocardial infarction and one patient due to sepsis.

Postoperative complications were seen in 52 patients (15.5%). There was no significant difference in the rate of postoperative complications between the groups (16.4 versus 14.4%) (Table 4).Clinical anastomosis leak was seen in three patients, and one patient was re-operated. In addition, two other patients needed re-operation due to postoperative bleeding and stomal stricture.

**Table 4:** Postoperative complications

	Preop. CRT (*n* = 177)	Postop. CRT (*n* = 159)
Wound infection (*n*)	7	4
Anastomotic leak	3 (1 op.)	–
Intraabd. abscess	–	1 (PCD)
Pelvic abscess	1	–
UTI	3	4
Pneumonia	2	3
Atelectasis	4	6
CVE	3	2
Postop. bleeding	2	1 (op.)
Prerenal azotemia	3	2
Stoma stenosis	1 (op.)	–
Re-operation	2	1
Total *n* (%)	29 (16.4)	23(14.4)

*UTI* urinary tract infection, *CVE* cardiovascular event, *PCD* percutaneous catheter drainage

### Chemoradiotherapy-dependent acute and chronic toxicity

Grade V toxicity developed in four patients (1.2%) following acute toxicity. Three patients in the preoperative group died during adjuvant CT (two patients due to neutropenic sepsis and one patient due to pancreatitis). One patient in the postoperative group also died during CRT (due to neutropenic sepsis).

Grade II, III, and IV acute toxicities were seen in a total of 130 patients (39%). There was no significant difference in the rate of toxicities between the groups (40 versus 39%). However, Grade III gastrointestinal system (GIS) toxicity was slightly lower in the preoperative group (6.9 versus 16.1%) (Table 5).

**Table 5:** Acute and late toxicity due to CRT

	ACUTE		LATE	
	Preop. CRT (*n* = 177)	Postop. CRT (*n* = 159)	Preop. CRT (*n* = 177)	Postop. CRT (*n* = 159)
Grade II	46	33	N/A	N/A
Grade III	19	29	12	17
Grade IV	2	1	1	4
Grade V	3	1	–	–
Total	70	64	13	21
Grade III gastrointestinal system toxicity				
Diarrhea (IV replacement)	10	24	–	–
Pad usage	2	2	–	–
Obstruction (need surgery)	–	–	2	5
Anast. stenosis	–	–	5	9
Grade III genitourinary system toxicity				
Pollakuria (hourly)	1	–	–	–
Disuria, pelvic pain	1	–	–	–
Obst. üropathy-hydronephrosis	–	–	5	3
Grade III hematologic toxicity				
Platelets 25 < 50	1	1	–	–
WBC 1.0 < 2.0	1	2	–	–
Neutrophils 0.5 < 1.0	3	–	–	–
Grade IV gastrointestinal system toxicity				
Obstr.	1	–	–	–
Perforation	–	–	–	1
Colovesical fistula	–	–	–	1
Rectovesical—vaginal fistula	–	–	–	2
Enterocutaneous fistula	–	–	1	–
Grade IV hematologic toxicity				
WBC < 10	1	1	–	–

*Preop* preoperative, *Postop* postoperative, *Obstr* obstruction, *WBC* leukocyte, *Anast.* anastomotic

In terms of late toxicity, only Grade III and IV toxicities were evaluated. Grade III–IV total toxic effects were significantly lower in the preoperative group (7.4 versus 13.2%, *p* = 0.002). In terms of Grade III genitourinary system (GUS) toxicities, eight patients had urethral obstruction and hydronephrosis, whereas fistulas were detected as Grade IV GIS toxicities (Table 5).

### Events during follow-up

As of January 2017, 248 (73.5%) survivors were followed for a mean of 60.4 (range 2 to 168) months. The duration of follow-up was > 4 years in 58% patients and > 5 years in 44% patients.

Except for three patients with postoperative mortality, a total of 85 deaths (25.3%) occurred during follow-up. Fifty-three cases were related to rectal cancer (disease progression, *n* = 49; treatment-related, *n* = 4), 27 to other causes, and five to secondary non-rectal cancers.

LR was seen in 36 patients (10.7%). Ten patients (2.9%) had isolated pelvic recurrence, and 24 patients (7.7%) had isolated pelvic recurrence and distant metastasis. In addition, 13 patients (4%) had second primary cancers and five patients (1.5%) had metachronous colon cancer.

### Locoregional recurrence (LR) and distant recurrence (DR)

**Fig. 2:**
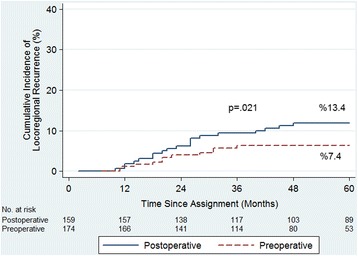
Cumulative incidence of locoregional recurrences among the 336 patients prospectively assigned to preoperative or postoperative chemoradiotherapy

LR was seen in a total of 12 patients (isolated pelvic recurrence, *n* = 3) in the preoperative group and in 24 patients (isolated pelvic recurrence, *n* = 7) in the postoperative group. Five-year cumulative LR rate was 7.4% in the preoperative group and 13.4% in the postoperative group. There was a significant difference in the competing-risk regression analysis (*p* = 0.021; hazard ratio [HR] 2.258; 95% confidence interval [CI] 1.128–4.520; Fig. 2).

Univariate and multivariate Cox regression analyses showed that R resection was the only significant prognostic factor affecting LR in the preoperative group (*p* = 0.002; HR 6.828; 95% CI 2.041–22.847). In the postoperative group, performance of MVR (*p* < 0.001), histopathological type (mucinous tumor; *p* = 0.024), T category (T4b; p = 0.021), LVI identification (*p* = 0.047), and disease stage (2c, *p* = 0.003) were found to be significant factors in the univariate analysis; however, MVR was the only most significant prognostic factor (*p* < 0.001) in the multivariate analysis (HR 17.213; 95% CI 3.846–77.029), followed by LVI identification (*p* = 0.034; HR 3.603; 95% CI 1.088–8.289).

Isolated distant metastases were observed in 19 patients in the preoperative group and in 29 patients in the postoperative group. There was no significant difference in the 5-year cumulative incidence between the groups (13.6 versus 18.4%; *p* = 0.071; HR 1.80; 95% CI 0.954–3.041).

### Cancer-specific survival (CSS)

**Fig. 3:**
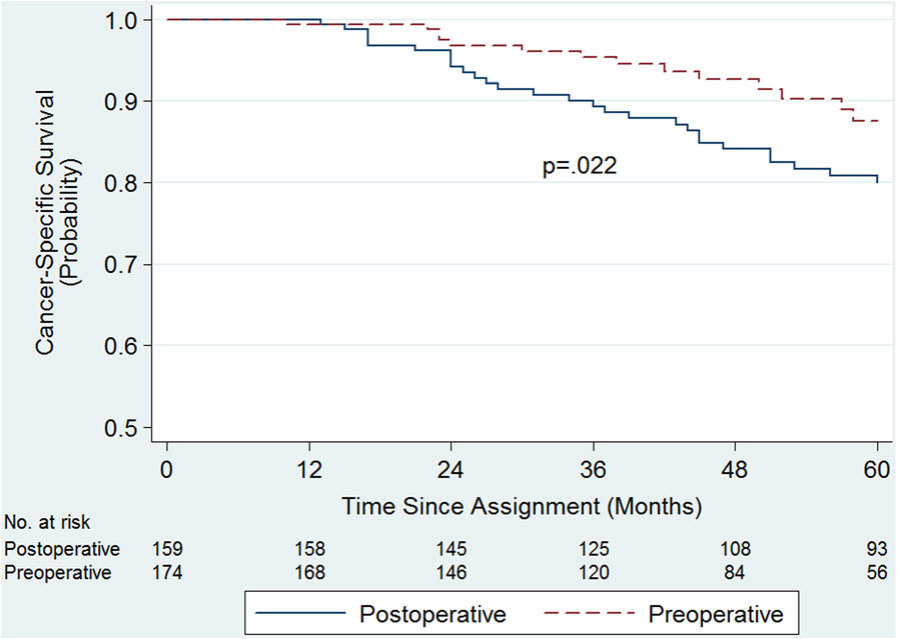
Cancer-specific survival among the 336 patients prospectively assigned to preoperative or postoperative chemoradiotherapy

Cancer-related mortality was seen in 16 patients in the preoperative group and in 42 patients in the postoperative group. Five-year CSS was significantly higher (87.5%) in the preoperative group, compared to the postoperative group (80%) (*p* = 0.022; HR 1.958; 95% CI 1.090–3.517) (Fig. 3).

In the preoperative group, univariate analysis showed that N-state (*p* = 0.008) and satellite tumor presence (*p* = 0.019) were significant prognostic factors affecting CSS. Multivariate analysis showed that N-state (N2b, N1B) was the most significant factor (*p* = 0.026; HR 11.148; 95% CI 13.38–92.909). In the postoperative group, univariate analysis showed that tumor localization (*p* = 0.004), performance of MVR (*p* = 0.012), operation type (*p* < 0.001), disease stage (*p* = 0.007), PNI identification (p = 0.007), and LVI identification (p < 0.001) were significant prognostic factors. Multivariate analysis also showed that operation type was the most significant prognostic factor (APR, *p* < 0.001; HR 9.857; 95% CI 2.767–35.122), followed by the presence of LVI (*p* = 0.001).

### Overall survival (OS)

Twenty-seven patients in the preoperative group and 58 patients in the postoperative group died due to various reasons. Five-year OS rate was higher in the preoperative group (79.8%), compared to the postoperative group (74.7%). A remarkable increase in the OS was seen (HR 1.558; 95% CI 0.974–2.493), although it did not reach statistical significance (*p* = 0.064).

### Disease-free survival (DFS)

Relapse was seen in 38 patients (distant metastasis, *n* = 19; LR, *n* = 12; second primary malignancy, *n* = 6; metachronous colon cancer, *n* = 1) in the preoperative group. In the postoperative group, relapse was seen in 64 patients (distant metastasis, *n* = 29; LR, *n* = 24; second primary malignancy, *n* = 7; metachronous colon cancer, *n* = 4). Although not statistically significant (*p* = 0.062), 5-year DFS was considerably higher in the preoperative group (75.2 versus 64.8%; HR 1.479; 95% CI 0.976–2.239).

## Discussion

This trial has shown that the preoperative administration of long-term CRT significantly prolonged CSS, compared to postoperative administration, suggesting a trend toward improved OS and DFS. We also confirmed that preoperative treatment significantly reduced the rates of locoregional recurrence and long-term toxic effects. To the best of our knowledge, this prospective trial is the first to demonstrate a significant improvement in survival rates and LR with preoperative long-term multimodality therapy, compared to postoperative treatment.

In the current study, 5-year cumulative LR rate was significantly lower in the preoperative CRT group (7.4 versus 13.4%; *p* = 0.021). This finding is consistent with several randomized studies, indicating a significant decrease in the LR rates as a result of RT addition to the treatment [7–13]. However, there are two prospective, randomized studies similar to our protocol, and only in the German study [1], 5-year cumulative LR incidence was significantly lower in the preoperative CRT group (6 versus 13%, *p* = 0.006). The LR rate in the current study, although slightly higher, is consistent with the German study. Given the similarities in RT dose, timing, CT regiments, and surgical method (necessity of TME), it is not surprising that LR rates were consistent in both studies. Another prospective, randomized study has shown identical LR rates (10.7%) between the groups, which is higher compared to our study [3]. This outcome is probably a result of performing TME occasionally rather than a routine protocol.

Among prognostic factors affecting LR (multivariate Cox regression analyses) in the present study, incomplete resection (R_1_) was found significant in preoperative CRT and performance of MVR and LVI (+) identification were found significant in the postoperative CRT group. R0 resection is a very important determinant of LR. In particular, several studies have shown that circumferential resection margin (CRM) positivity is an important prognostic factor of LR development [1, 13–18]. In conclusion, since tumor oxygenation is better with preoperative treatment than with postoperative treatment, irradiation seems to be more effective within preoperative settings [1, 10, 18]. In addition, higher R_0_ resection rates and compliance to CT in preoperative group which potentialize the effects of radiation are the other important factors increasing local success.

In rectal cancers, cancer-related mortality occurs depending on LR and/or distant metastasis development. Although there was no significant difference in isolated distant metastasis development between groups, the incidence was slightly lower in the preoperative group (13.6 versus 18.4%, *p* = 0.071). This is primarily due to the high level of patient compliance in the preoperative group. In addition, there is a possibility of more effective treatment of metastases following an earlier systematic treatment. There was a significant increase in CSS rates as a result of decreased LR and distant metastasis rates, probably, in the preoperative group.

On the other hand, we found no significant differences in CRT-dependent acute toxicity (Grade I–IV) between the groups. All cases of Grade V toxicity in the preoperative CRT group emerged during adjuvant CT. Although there was a significant decrease in LR rates as a result of concurrent CT to the preoperative radiotherapy [16, 17], prospective, randomized studies and meta-analyses showed that toxic effects increased and OS-DFS advantage was not provided particularly following adjuvant fluoropyrimidine-based CT after neoadjuvant CRT [19–21]. In our opinion, adjuvant CT might cause overtreatment in cases that respond very good to preoperative CRT, and the clinical benefit of this approach should be questioned. In our previous study which was comparing the preoperative RT alone, due to older age and associating comorbidities, versus preoperative CRT, there were not any statistically significant difference in disease recurrence and survival rates between the groups. Preoperative RT alone was mentioned as a good alternative approach for these kind of patients in that study [22].

In this study, late toxicity (Grade III–IV) was significantly low in the preoperative group (*p* = 0.002). It can be seen that preoperative CRT group was more advantageous, particularly in terms of Grade III–IV GIS toxicity (Table 5). In the German study, late toxic effects (Grade III–IV) and anastomotic strictures were significantly low in the preoperative group (*p* = 0.003). In the current study, five patients in the preoperative group and three patients in the postoperative group had retroperitoneal fibrosis-related obstructive uropathy-hydronephrosis for Grade III GUS toxicity (bilaterally developed in three patients and resulted in loss of function in two patients). Regarding this complication which is not present in literature except for one study [23], reporting Grade IV ureteral stricture, patients need to be monitored carefully and prevented from loss of function by urgently intervening when identified. In addition, gastrointestinal fistulas represent another major complication, which are seen in the current study, and has been only reported in the Stockholm studies [24, 25].

Nonetheless, the preoperative and postoperative CRT groups were not randomized, which represents the main limitation of the current study. This choice was based on the fact that we considered it would be more suitable to make the final decision about the treatment type together with the patient. The second limitation is the inadequate sample size to reach a significant statistical power. All surgeries were carried out by a single surgeon, thus resulting in the small sample size. During study planning, TME was not a standardized and commonly used technique in Turkey; therefore, the main concern was that patient prognosis could be influenced by different surgeons and techniques.

## Conclusions

In conclusion, long-term preoperative CRT in LARC is superior compared to postoperative treatment, in terms of patient compliance, late toxic effects, recurrence, and survival rates (CSS). However, a multidisciplinary approach by experienced teams and a curative TME particularly by an experienced colorectal surgeon are highly important for the success of treatment.

## Abbreviations

CI: Confidence interval
CRM: Circumferential resection margin
CRT: Chemoradiotherapy
CSS: Cancer-specific survival
CT: Chemotherapy
CTV: Clinical target volume
DFS: Disease-free survival
GIS: Gastrointestinal system
GTV: Gross tumor volume
GUS: Genitourinary system
Gy: Gray
HR: Hazard ratio
LARC: Locally advanced rectal cancer
LR: Locoregional recurrence
LVI: Lymphovascular invasion
MVR: Multivisceral resection
OS: Overall survival
PNI: Perineural invasion
PTV: Planning target volume
RT: Radiotherapy
TME: Total mesorectal excision
WHO: World Health Organization
